# Changing Trends in Phthalate Exposures: Zota and Woodruff Respond

**DOI:** 10.1289/ehp.1408629R

**Published:** 2014-10-01

**Authors:** Ami R. Zota, Tracey J. Woodruff

**Affiliations:** 1 Department of Environmental and Occupational Health, Milken Institute School of Public Health, George Washington University, Washington, DC, USA; 2 Program on Reproductive Health and the Environment, Department of Obstetrics, Gynecology, and Reproductive Sciences, University of California, San Francisco, Oakland, California, USA

We appreciate Lioy et al.’s interest in our recent article (Zota et al. 2014) documenting temporal trends in phthalate exposures in the U.S. general population. Lioy et al. raise important points about the future implications of our findings.

We agree that the downward trend in exposures to phthalates such as di-*n*-butyl phthalate (DnBP) and di(2-ethylhexyl) phthalate (DEHP) is encouraging, but the rising trend in other phthalates, such as diisobutyl phthalate (DiBP) and diisononyl phthalate (DiNP), is worrisome. Lioy et al. note that DiNP is less potent as an antiandrogen than the other phthalates. However, its increasing presence in the U.S. general population warrants public health concern: The U.S. Environmental Protection Agency (EPA) has expressed concern about DiNP’s use [Phthalates Action Plan (U.S. EPA 2012)]; DiNP was added to California’s Proposition 65 List of Potential Carcinogens in 2013 (California Office of Environmental Health Hazard Assessment 2014); and it can act cumulatively with other phthalates to affect male reproductive end points (National Research Council 2008). Given the toxicological evidence of potential harm, further study on its adverse health effects in epidemiologic studies is warranted.

Lioy et al. also comment on the importance of studying phthalate substitutes. We agree that it is important for NHANES (National Health and Nutrition Examination Survey) and other large environmental health studies to evaluate the presence of potential “substitute” chemicals not only of phthalates but also of other environmental chemicals to provide the best human exposure information. In fact, for the most recent NHANES survey (2011–2012), scientists at the Centers for Disease Control and Prevention are beginning to release population-level data for the urinary metabolite of 1,2-cyclohexane dicarboxylic acid diisononyl ester (DINCH), a nonphthalate plasticizer that is used as a replacement for some of the high-molecular-weight phthalates (Centers for Disease Control and Prevention 2013). Indeed, biomonitoring data are extremely valuable for understanding population exposures to high production volume chemicals and helping to prioritize chemicals of concern. However, NHANES records only the actions of the past. To fully characterize public health risks of chemicals and develop effective exposure-reduction strategies, we need greater disclosure from manufacturers on chemical ingredients in consumer and other products; more complete data from the U.S. EPA on chemical production and use; and data to evaluate potential health hazards from these chemicals. With this information, we can make informed decisions about the use of chemicals in the market place, limit those that pose a risk to the population, and improve the public’s health.
